# The Major Source of Antioxidants Intake From Typical Diet Among Rural Farmers in North-eastern Japan in the 1990s

**DOI:** 10.2188/jea.JE20190237

**Published:** 2021-02-05

**Authors:** Megumi Tsubota-Utsugi, Jun Watanabe, Jun Takebayashi, Tomoyuki Oki, Yoshitaka Tsubono, Takayoshi Ohkubo

**Affiliations:** 1 Department of Hygiene and Preventive Medicine, Iwate Medical University School of Medicine, Iwate, Japan; 2 Food Research Institute, National Agriculture and Food Research Organization, Ibaraki, Japan; 3 Department of Food Function and Labeling, National Institute of Health and Nutrition, National Institutes of Biomedical Innovation, Health and Nutrition, Tokyo, Japan; 4 Department of Nutritional Sciences, Graduate School of Nutritional Sciences, Nakamura Gakuen University, Fukuoka, Japan; 5 Department of Global Health, Graduate School of Economics, Tohoku University, Miyagi, Japan; 6 Department of Hygiene and Public Health, Teikyo University School of Medicine, Tokyo, Japan

**Keywords:** antioxidant capacity, dietary records, Japanese, oxygen radical absorbance capacity (ORAC) method, overall diet

## Abstract

**Background:**

Previous Japanese studies have led to the erroneous conclusion of antioxidant capacity (AOC) intakes of the overall Japanese diet due to limitations in the number and types of food measured, especially in rice and seafood intake. The aims of the study were to construct an AOC database of foods representative of the typical Japanese diet and to clarify the high contributors to AOC intake from the overall diet of the Japanese population.

**Methods:**

Commonly consumed foods were estimated using 3-day dietary records (DRs) over the four seasons among 55 men and 58 women in Japan. To generate an AOC database suitable for the typical Japanese diet, hydrophilic (H-)/lipophilic (L-) oxygen radical absorbance capacity (ORAC) values of foods in each food group were measured via validated methods using the food intake rankings. Subsequently, we estimated the AOC intake and the AOC characteristics of a typical Japanese diet.

**Results:**

Of 989 food items consumed by the participants, 189 food items were measured, which covered 78.8% of the total food intake. The most commonly consumed types of antioxidant-containing food were tea, soybean products, coffee, and rice according to H-ORAC, and soybean products, fish and shellfish, vegetables, and algae according to L-ORAC.

**Conclusions:**

The characteristics of high AOC intake in rice and seafood more appropriately reflected the Japanese-style diet. Further studies are expected to clarify the association between food-derived AOC and its role in preventing or ameliorating lifestyle-related diseases.

## INTRODUCTION

There is increasing awareness regarding the health risks of reactive oxygen species in the body; however, the ability of the antioxidant capacity (AOC) to prevent or ameliorate various chronic diseases remains controversial.^1^^–^^7^ There are several possible explanations for these discrepancies. First, high AOC intake from a certain food does not necessarily translate to a large contribution of the food to the overall diet. It is important to consider not only the AOC levels per gram of specific foods but also their total contribution to AOC of the typical diet. Second, even though previous studies reported that AOC data was collected using the “oxygen radical absorbance capacity (ORAC) method,” which is one of the most widely used methods to evaluate AOC,^8^ there was large variation in the methods employed in the measurement process. Furthermore, the AOC values derived from hydrophilic ORAC (H-ORAC) and lipophilic ORAC (L-ORAC) in previous epidemiological studies were often used interchangeably.^1^^,^^9^^–^^15^ Generally, water-soluble antioxidants suppress cytoplasmic substrate and plasma oxidation, whereas lipid-soluble antioxidants prevent lipid peroxidation in the cell membranes.^16^ Considering that AOC for scavenging reactive oxidants does not always correlate linearly with the capacity to inhibit oxidation of biological molecules^17^ and AOC in food changes during uptake and metabolism,^18^ the efficacy of antioxidants may depend on concentrations, localizations, physiological mobilities, and interactions of oxidants and/or antioxidants. In fact, lipophilic components have been confirmed to have different functions and/or metabolic pathways in the body due to differences in the physicochemical property of hydrophilic components.^19^ In addition, the plasma concentrations of H-ORAC and L-ORAC after meals varied due to differences in distribution, metabolism, and clearance.^19^ These differences may have caused the prevention of cognitive impairment only when using a lipophilic antioxidant supplement based on a previous study.^20^ In terms of disease prevention, the effects of peroxyl radial-scavenging activities of water-soluble and lipid-soluble antioxidants in foods may differ because of different pharmacokinetics.

Moreover, it is known that geographic location and growing conditions can affect the AOC of foods^21^; despite this, previous Japanese studies referenced AOC values from Western countries,^9^^–^^15^ so the AOC values of foods with high intake in the Japanese population, such as rice and seafood, which were previously thought to exist at very low levels or not at all, or foods not regularly consumed in Western countries, were not adequately measured. It is considered that foods with a high antioxidant contribution in Japanese diet are more likely to be contained in foods that have not been previously measured foods in other countries. Using an appropriate AOC database that reflects the overall Japanese diet and identifying the effects of Japanese diet with health conditions will help to elucidate the relationships between the AOC intake and health outcomes in epidemiological studies. We, therefore, aimed to construct a hydrophilic and lipophilic AOC database of foods representative of the Japanese diet employing dietary records (DRs) and a validated H-/L-ORAC method, and to identify the high contributors to AOC from the overall Japanese diet. This part of the project elucidates the relationship between antioxidant intake from a habitual diet and disease outcomes in the general population; the present report provides basic data about the amount of antioxidant intake in the Japanese diet that can be used in future prospective studies.

## MATERIALS AND METHODS

**Figure 1.**
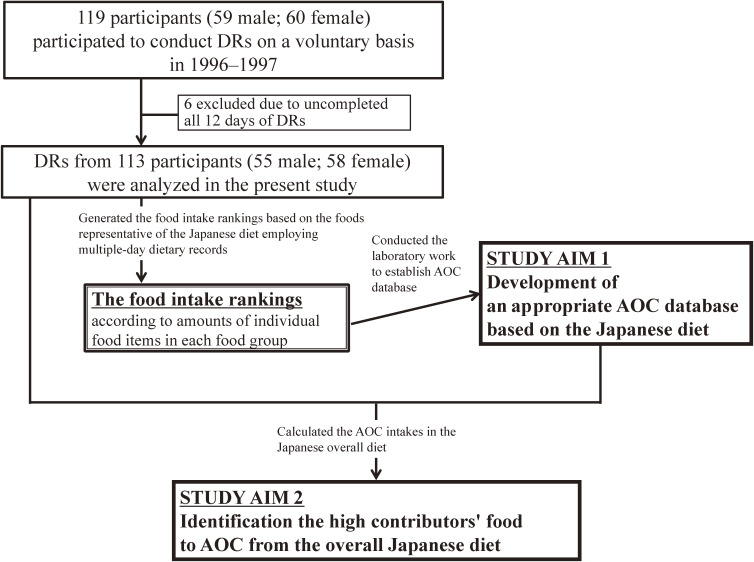
Flow flowchart of the present study. AOC, antioxidant capacity; DRs, dietary records.

Figure 1 presents a flowchart of the present study. To construct an AOC database representative of the Japanese diet, we generated food intake rankings of food groups with the highest consumption by Japanese and those most commonly marketed in Japan using multiple-day DRs in a Japanese population.^22^ A total of 59 men and 60 women were selected on a voluntary basis in Miyagi Prefecture in the northern part of Japan. In this study, we adhered to the code of ethics at the time of the survey in 1995. This noninvasive observational study complies with the Declaration of Helsinki, and written informed consent was obtained from all of the participants before the investigation. The data we used was already anonymized at the institution, and personal information cannot be identified.

### Multiple-day DRs

DRs were collected using the 3-day DR as a reference method during each of the four seasons over a period of 1 year: in the month of November (autumn) of 1996 and in February (winter), May (spring), and August (summer) of 1997.^23^^,^^24^ The participants were instructed to record all food items and beverages consumed in a standardized booklet. They were asked to provide a detailed description of each food item (open ended), including the weight of food prepared and the proportion consumed. Research dietitians assessed the records in a standardized manner after completion by the participants and calculated intake of all food items and beverages using the Japanese Standard Food Composition Table 2010.^25^ If codes were not available for certain local foods, the dietitian substituted the food considered to be most similar by asking subjects for details about the food. Next, we generated a list ranked according to amounts of individual food items in each food group.

### Selection of food items for inclusion in the AOC database

Based on the ranking in the list, we measured food items for inclusion in the AOC database using the following standards: (1) selecting high-intake food from each food group; (2) all food items were commercially purchased in local grocery stores in Japan; (3) two or more items, with some exceptions, for each food item that were independently obtained in different regions and harvest seasons; and (4) using the same analytical method to reliably measure the H/L-ORAC in multiple laboratories. AOC data on plant-derived foods were described separately,^21^^,^^26^^,^^27^ and the remainder, including animal-derived foods, was newly measured. eTable 1 shows details of the referenced AOC values of food items in the present study.

### H- and L-ORAC measurement

The ORAC assays were based on the standardized methods described previously.^28^^,^^29^ The ORAC assay enables measurement of radical-scavenging capacity against peroxyl radicals generated by the thermo-degradation of 2,2′-azobis(2-amidinopropane) dihydrochloride.^8^ H-/L-ORAC values were measured separately. The H-ORAC assay was performed by evaluating the antioxidant capacities of acidified aqueous methanol (methanol:water:acetic acid = 90:9.5:0.5, MWA) extracts,^28^ and the L-ORAC assay was performed on n-hexane/dichloromethane (1:1) extracts.^29^ These methods were subjected to inter-laboratory tests and had been validated.^28^^–^^30^ An MWA solution of ferulic acid (1 mg/mL) was used as a quality control, and the confirmed H-ORAC value of the solution was 17,552 ± 1,864 µmol TE/L (mean ± 2S_R_ [reproducibility standard deviation]) in the multi-laboratory validation study. A dimethyl sulfoxide (DMSO) solution of Trolox (800 mg/L) was used as a positive control for L-ORAC measurements, and DMSO without an antioxidant was utilized as a negative control to ensure the reliability of each measurement. All data were expressed as micromoles of Trolox equivalents per gram (µmol-TE/g edible portion). The H-ORAC and L-ORAC measurements of the selected food items were performed in three laboratories. All laboratories participated in the interlaboratory tests for the validation of H-ORAC and L-ORAC measurements. The reproducibility relative standard deviations of the H-ORAC and L-ORAC measurements ranged from 4.4% to 13.8% and from 14.8% to 19.4%, respectively.

### Statistical analysis

First, the sum of AOC intake for total and individual food groups was computed to estimate the contributions of H-/L-ORAC values. The proportion of AOC-containing foods, relative to total food consumption, was calculated as follows: weight (g/day) of each food group with measured AOC consumed × 100/weight (g/day) of each food group consumed. To assess AOC intake in the overall diet, the characteristics of daily food intake (g/day) as well as the amounts of H- and L-ORAC intake in the 12 days of DRs were estimated.

Next, to examine the distribution of dietary characteristics across quartiles of each total-ORAC, H-ORAC, and L-ORAC, we generated a general linear model of each food group. The correlation between H-ORAC and L-ORAC was measured using Pearson’s Correlation Coefficient. Then, we calculated the ratios of within-person and between-person variance.^31^

All analyses were performed using SAS software (ver. 9.4; SAS Institute Inc., Cary, NC, USA).

## RESULTS

In this study, 113 participants (55 men and 58 women) who completed all 12 days of dietary records were used in the analysis. The age range was 45–77 (mean, 62) years, and the majority of participants were farmers, self-employed, or housewives.

Table 1 presents the number of food items and measured antioxidant capacity in the present study. The number of food items consumed by the participants totaled 989. Of these, 189 food items were subjected to determination of ORAC values.

**Table 1.** Number of food items consumed by participants and measured antioxidant capacity in the present study

Food groups	Number of food items		

	Presented in the Standard Tables of Food Composition in Japan 2010	Consumed by the participants	Measured AOC in the present study
Rice, bread, and noodles	138	56	12
Potatoes	40	21	5
Sugars	23	18	5
Beans	73	36	9
Nuts and seeds	37	19	6
Vegetables	326	150	29
Fruits	157	78	22
Mushrooms	36	24	5
Algae	47	32	7
Fish and shellfish	388	214	21
Meats	244	92	14
Eggs	20	12	4
Dairy products	52	32	7
Fat and oil	22	12	3
Confectioneries	120	90	12
Beverages	55	37	12
Seasonings and spices	84	54	11
Prepared foods	16	12	5

Total	1,878	989	189

AOC, antioxidant capacity.

Table 2 displays the total food intake (g/day) and AOC (µmol TE/day) in the typical diet. The proportion of AOC-containing foods in the AOC measurement, which is relative to the total food consumption, was 78.8%, and those for seasonings and spices as well as eggs were 48.1% and 98.8%, respectively. The estimated total ORAC intake was 14,600 µmol TE/day, with 13,300 µmol TE/day according to H-ORAC and 1,360 µmol TE/day according to L-ORAC. The major contributors to AOC intake according to food group were beverages (46.2%), followed by vegetables (10.7%), grain products (8.9%), beans (8.7%), and fruits (6.8%) for H-ORAC; and fish and shellfish (27.2%), followed by seasoning and spices (21.6%), beans (14.7%), vegetables (11.6%), and eggs (5.8%) for L-ORAC. The majority of H-ORAC intake was of plant origin, whereas about 60% of L-ORAC intake was plant derived.

**Table 2.** Total food intake (g/day) and antioxidant capacity (µmol TE/day) in the study participants

Food groups	Food intake (g/day)	Weight contribution to AOC intake (%)	H-ORAC		L-ORAC	
	
			Intake (µmol TE/day)	AOC-containing foods/total food consumption (%)	Intake (µmol TE/day)	AOC-containing foods/total food consumption (%)
Rice, bread, and noodles	533.7	93.1	1,184.6	8.9	38.4	2.8
Potatoes	55.1	86.3	202.5	1.5	26.1	1.9
Sugars	9.8	97.8	3.2	0.0	0.1	0.0
Beans	87.3	90.3	1,156.6	8.7	198.8	14.7
Nuts and seeds	2.8	96.5	72.0	0.5	18.6	1.4
Vegetables	266.5	64.1	1,411.8	10.7	156.8	11.6
Fruits	133.5	76.7	895.4	6.8	31.2	2.3
Mushrooms	11.5	80.6	32.9	0.2	15.3	1.1
Algae	13.5	87.0	132.8	1.0	33.1	2.4
Fish and shellfish	126.8	55.2	395.3	3.0	369.7	27.2
Meats	44.4	67.9	82.8	0.6	42.6	3.1
Eggs	44.9	98.8	344.9	2.6	78.2	5.8
Dairy products	158.8	95.2	236.2	1.8	4.3	0.3
Fat and oil	9.2	91.8	4.4	0.0	NQ	NQ
Confectioneries	32.4	78.9	143.1	1.1	43.5	3.2
Beverages	700.8	91.8	6,119.0	46.2	ND	ND
Seasonings and spices	67.0	48.1	806.7	6.1	292.7	21.6
Prepared foods	5.7	95.7	28.7	0.2	7.5	0.5

Total	2,304.0	78.8	13,252.9	100.0	1,357.0	100.0

AOC, antioxidant capacity; H-ORAC, hydrophilic oxygen radical absorbance capacity; L-ORAC, lipophilic ORAC; ND, not determined; NQ, not quantitated.

Table 3 presents the top 30 food items that contributed to high AOC intake in the study participants. The most commonly consumed types of food were green tea (32.1%), rice (7.0%), coffee with milk (5.8%), *natto* (5.4%), and *miso* (4.4%) for H-ORAC; and *miso* (19.4%), skipjack tuna (5.5%), deep-fried *tofu* (5.5%), egg (5.3%), and *natto* (5.3%) for L-ORAC. Notably, the 30 food items in Table 3 represent 85.9% of H-ORAC and 80.1% of L-ORAC intake.

**Table 3.** The top 30 food items that contributed to high antioxidant capacity intake in the study participants

TOP	H-ORAC				L-ORAC			
	
	Food item number^a^	Food and description	Intake (µmol TE/day)	AOC-containing foods/total food consumption (%)	Food item number^a^	Food and description	Intake (µmol TE/day)	AOC-containing foods/total food consumption (%)
1	16,037	Green tea, sencha, infusion	4,255.6	32.1	17,046	Rice-koji miso, red type (*miso*)	262.9	19.4
2	1,088	Cooked paddy rice, well-milled rice (*rice*)	932.7	7.0	10,087	Skipjack, caught in autumm, raw	75.0	5.5
3	16,047	Coffee drink containing milk	772.3	5.8	4,040	Soybean, abura-age (*deep-fried tofu*)	74.7	5.5
4	4,046	Soybean, itohiki-natto (*natto*)	713.7	5.4	12,004	Hen’s egg, whole, raw	72.3	5.3
5	17,046	Rice-koji miso, red type (*miso*)	585.8	4.4	4,046	Soybean, itohiki-natto (*natto*)	71.3	5.3
6	16,006	Beer, pale	465.9	3.5	10,345	Japanese common squid (*surumeika*), raw	67.7	5.0
7	7,148	Apple, raw	438.5	3.3	10,173	Pacific saury, raw	42.1	3.1
8	16,045	Coffee, infusion	327.5	2.5	10,202	Walleye pollack, roe (*tarako*), raw	29.6	2.2
9	12,004	Hen’s egg, whole, raw	326.2	2.5	10,045	Japanese anchovy, niboshi (*niboshi*)	28.7	2.1
10	6,191	Eggplant, fruit, raw	323.8	2.4	1,015	Wheat, soft flour, first grade	27.6	2.0
11	6,084	Edible burdock, root, raw	270.9	2.0	10,253	Bluefin tuna, Lean meat, raw	26.6	2.0
12	4,032	Soybean, momen-tofu (*tofu*)	166.1	1.3	4,033	Soybean, kinugoshi-tofu (*tofu*)	25.6	1.9
13	4,033	Soybean, kinugoshi-tofu (*tofu*)	160.7	1.2	4,032	Soybean, momen-tofu (*tofu*)	21.6	1.6
14	16,042	Oolong tea, infusion	150.5	1.1	10,205	Pacific cod, raw	21.0	1.5
15	2,017	Potato, tuber, raw	142.5	1.1	10,100	Brown sole, raw	20.7	1.5
16	6,153	Onion, bulb, raw	139.6	1.1	6,139	Japanese radish (*daikon*), takuan-zuke	18.9	1.4
17	6,134	Japanese radish (*daikon*), root without skin,	129.3	1.0	2,017	Potato, tuber, raw	18.5	1.4
18	7,012	Strawberry, raw	109.5	0.8	6,061	Cabbage, head, raw	17.1	1.3
19	6,182	Tomato, fruit, raw	93.0	0.7	6,201	Turnip rape, flower buds and stems, raw	16.1	1.2
20	6,201	Turnip rape, flower buds and stems, raw	91.7	0.7	10,134	Salmon, chum salmon, raw	15.6	1.2
21	13,003	Ordinary liquid milk	91.5	0.7	17,045	Rice-koji miso, light yellow type (*miso*)	15.6	1.1
22	10,087	Skipjack, caught in autumm, raw	88.6	0.7	9,045	Wakame, blanched and salted, desalted	14.9	1.1
23	6,061	Cabbage, head, raw	82.7	0.6	11,186	Pork, Vienna sausage	14.8	1.1
24	4,040	Soybean, abura-age (*deep-fried tofu*)	82.5	0.6	6,207	Chinese chive, leaves, raw	14.6	1.1
25	9,004	Purple laver, toasted	78.4	0.6	6,065	Cucumber, fruit, raw	14.3	1.1
26	7,062	Grapefruit, juice sac, raw	77.6	0.6	15,116	Milk chocolate	14.1	1.0
27	13,005	Milk containing recombined milk, low fat	76.0	0.6	6,086	Komatsuna, leaves, raw	12.8	0.9
28	17,045	Rice-koji miso, light yellow type (*miso*)	74.2	0.6	6,182	Tomato, fruit, raw	11.5	0.8
29	16,039	Ban-cha, infusion	68.5	0.5	5,018	Sesame seed, roasted	10.7	0.8
30	7,136	Peach, raw	67.3	0.5	15,009	Kasutera	10.6	0.8

AOC, antioxidant capacity; H-ORAC, hydrophilic oxygen radical absorbance capacity; L-ORAC, lipophilic ORAC.

^a^Item numbers were addressed under the food composition table 2010.

Seasonal variation in food contributions to AOC intake is shown in eTable 2. The mean H-/L-ORAC intake for 3 consecutive days in each season was 13,100/1,340 µmol TE/day in spring, 12,900/1,380 µmol TE/day in summer, 13,700/1,350 µmol TE/day in autumn, and 13,300/1,360 µmol TE/day in winter. Even though the amount of food intake was the highest in a certain season, the ORAC intake values were not necessarily the highest in that season. The amount of fruit intake was highest in autumn (183 g/d); however, the contribution of fruit intake to the H-ORAC value was highest in winter (2,020 µmol TE/day), and that to the L-ORAC was highest in spring (72.8 µmol TE/day). Although seasonal differences were not observed for the intake of fish and shellfish and meat, the highest contributions to ORAC intake were observed in summer for fish and shellfish, and in autumn for meat.

The distribution of dietary characteristics across quartiles for total-ORAC, H-ORAC, and L-ORAC is shown in Table 4. Significant differences in food intake across quartiles of total-ORAC were found only for food groups with a high contribution to H-ORAC intake. Diets with high H-ORAC values were characterized by the high intake of nuts and seeds, vegetables, fruits, animal protein-rich foods, beverages, and seasonings and spices, whereas those with high L-ORAC values were characterized by the high intake of grain products, beans, vegetables, fish and shellfish, and seasonings and spices. There was a significant Pearson’s correlation coefficient between H-ORAC and L-ORAC (*r* = 0.39). On an individual basis, these amounts will vary considerably from average depending upon the number of foods consumed daily (data not shown). Further, intake showed greater intra-individual variance than inter-individual variance (average ratio of intra-/inter-individual variance, H-ORAC: 2.9 [range, 0.8–7.6]; L-ORAC: 3.5 [range, 1.3–7.4]).

**Table 4.** The distribution of dietary characteristics across quartiles for AOC in the study participants

	Quartile of total ORAC			Quartile of H-ORAC			Quartile of L-ORAC		
		
	1 (lowest)	4 (highest)	*P*-value^a^	1 (lowest)	4 (highest)	*P*-value^a^	1 (lowest)	4 (highest)	*P*-value^a^
ORAC intake, range, µmol TE/day	7,182.5–11,998.5	16,531.7–27,086.6		6,189.8–10,533.2	15,135.2–25,504.9		627.4–1,098.2	1,560.9–2,712.1	
Rice, bread, and noodles, g	478.4 (143.3)	568.6 (202.9)	*0.242*	467.8 (135.4)	563.3 (199.7)	*0.144*	471.8 (152.4)	615.5 (225.4)	0.014
Potatoes, g	54.3 (26.1)	57.8 (21.8)	*0.842*	55.1 (25.7)	57.5 (22.0)	*0.876*	51.9 (23.8)	63.1 (19.4)	0.098
Sugars, g	9.2 (4.6)	10.3 (6.7)	*0.311*	8.8 (4.4)	10.4 (6.6)	*0.529*	10.2 (5.2)	11.3 (6.1)	0.17
Beans, g	84.7 (29.8)	90.8 (38.1)	*0.911*	85.5 (31.2)	92.0 (36.7)	*0.819*	73.8 (30.1)	99.1 (30.7)	0.024
Nuts and seeds, g	1.7 (2.0)	3.7 (3.1)	*0.017*	1.7 (2.0)	3.6 (3.1)	*0.032*	2.1 (2.9)	3.7 (2.9)	0.105
Vegetables, g	225.2 (61.2)	291.3 (73.3)	*0.001*	226.4 (63.4)	290.0 (75.1)	*0.001*	222.2 (90.3)	304.1 (73.5)	0.003
Fruits, g	106.6 (59.2)	165.9 (75.0)	*0.007*	104.1 (59.7)	164.9 (75.5)	*0.004*	120.8 (46.6)	132.2 (71.3)	0.552
Mushrooms, g	9.0 (6.1)	12.4 (8.2)	*0.088*	8.7 (5.2)	12.2 (8.2)	*0.034*	10.3 (7.4)	12.5 (6.9)	0.673
Algae, g	12.5 (7.4)	13.7 (9.5)	*0.09*	12.4 (7.4)	14.0 (9.4)	*0.821*	10.3 (8.2)	14.6 (9.5)	0.136
Fish and shellfish, g	113.1 (33.9)	136.8 (39.9)	*0.001*	112.2 (31.6)	135.8 (39.2)	*0.067*	105.6 (31.5)	155.9 (42.2)	<0.001
Meats, g	38.2 (20.1)	52.3 (19.7)	*0.007*	37.8 (20.0)	52.2 (19.7)	*0.009*	38.2 (19.4)	49.0 (21.5)	0.314
Eggs, g	42.2 (22.7)	48.3 (17.3)	*0.224*	43.0 (22.8)	48.8 (17.9)	*0.467*	40.8 (15.7)	52.6 (20.4)	0.1
Dairy products, g	164.1 (86.2)	155.9 (119.3)	*0.864*	169.0 (82.6)	162.0 (116.4)	*0.728*	162.7 (99.8)	141.0 (111.9)	0.423
Fat and oil, g	8.8 (3.1)	10.6 (4.2)	*0.07*	8.7 (3.1)	10.7 (4.3)	*0.07*	8.2 (3.5)	10.0 (3.4)	0.223
Confectioneries, g	29.4 (27.2)	32.5 (27.3)	*0.89*	28.9 (27.2)	32.4 (27.3)	*0.817*	33.8 (26.7)	39.0 (25.2)	0.288
Beverages, g	368.1 (157.6)	1,074.0 (435.3)	*<0.001*	373.3 (165.5)	1,064.7 (431.0)	*<0.001*	626.0 (303.2)	756.0 (315.4)	0.542
Seasonings and spices, g	58.8 (15.5)	76.3 (20.1)	*<0.001*	58.1 (15.4)	76.2 (20.2)	*0.001*	56.5 (13.8)	75.7 (16.2)	<0.001
Prepared foods, g	6.3 (8.6)	7.5 (14.1)	*0.472*	6.0 (8.7)	6.1 (12.2)	*0.628*	5.0 (9.3)	5.0 (8.7)	0.842

AOC, antioxidant capacity; H-ORAC, hydrophilic oxygen radical absorbance capacity; L-ORAC, lipophilic ORAC.

Variables are presented as mean (standard deviation).

^a^Obtained using generalized linear model.

## DISCUSSION

To the best of our knowledge, this is the first study to elucidate AOC intake of the most commonly consumed and marketed foods in Japan, including rice and seafood, using multiple-day DRs in a Japanese population. The present study revealed that tea, rice, seafood, and soybean products, which are characteristic of the Japanese diet, showed the highest contributions to AOC.

Our study has several strengths. The sampling protocol attempted to take into account the potential variation that might exist in the Japanese market as well as reflect the Japanese diet of the consumer. Moreover, we employed a validated ORAC method, thereby confirming the method and allowing comparisons with values from other researchers. We thus expect generalizability of our developed AOC database to other Japanese studies.

In the present study, we measured the AOC values of 189/998 food items, which represented 78.8% of the total food intake. More than 60% of total AOC intake was represented by tea, soybean products, coffee, and rice according to H-ORAC, and soybean product, fish and shellfish, and vegetables according to L-ORAC. In contrast, previous study in Western countries using the FFQ revealed that more than 50% of AOC intake was derived from vegetables and fruits, followed by grains, tea, chocolate, and beverages.^32^ The present study also reported the contribution of vegetables and fruits to H-ORAC; however, tea, rice, soybean products, and fish, which are characteristic of the Japanese diet, were large contributors to AOC intake among Japanese. The present study seems to support our hypothesis that foods with a high antioxidant contribution in Japanese diet are more likely to be contained in foods that have not been measured foods in other countries until now. Our results, which revealed the high contribution of tea and beans, are in partial agreement with previous Japanese studies.^9^^–^^15^ However, these previous Japanese studies relied on AOC values from reports from Western countries and used not only analysis data but also substitution and calculation data. Due to limitations of the sampling protocol, these studies did not collect AOC values of grain products, soy products, and seafood, which are commonly consumed in Japan, and did not distinguish between H-ORAC and L-ORAC values. Consequently, researchers came to the erroneous conclusion that these foods did not exhibit AOC, or contributed minimally to AOC values in the Japanese diet.^9^^–^^15^ Compared to previous Japanese studies, ORAC values in beans, fruits, and potatoes were slightly lower in the present study; however, the total amount of such food intake was similar to that reported previously.^10^ It is likely that differences in the environmental conditions (eg, climate, location, and soil fertility) of the collected foods and the measurement methods employed are the reasons for these differences.^33^^,^^34^

We found that seasonings and spices, especially seasonings with soybean products, such as miso made high contributions to AOC. Previous studies reported extremely high H- and L-ORAC values for certain spices and herbs, indicating that the component essential oils contained considerable quantities of antioxidants.^35^ However, the AOC contribution from herbs is not high in Japan. It is important to note that, in surveys of dietary behavior and consumption, the nutrient intake values are usually computed from the raw weight of food items before cooking and processing, and not from the actual quantity consumed. In fact, several food items, such as fish (eg, Japanese anchovy [*niboshi*]; skipjack tuna) processed products (eg, dried fillet of bonito [*katsuo-bushi*]), shellfish (eg, Japanese corbicula clam [*shijimi*]), and algae (eg, dried giant kelp [*konbu*]), are only used for preparing soup stock and are not directly consumed.^36^ Thus, the contribution of seasonings to ORAC intake might be slightly overestimated compared to the actual intake. On the other hand, many of dishes using miso are miso soup. Therefore, actual consumption of miso is considered relatively high, unlike other seasonings. According to the ORAC values, seasonings and spices are excellent antioxidant sources; however, it is difficult to estimate typical amounts consumed because they are used in relatively small quantities in recipes and formulations.

We found seasonal differences in the AOC values of food groups. Japan has four distinct seasons, and the Japanese tend to prefer seasonal foods, such as salmon, young sardines, and wild vegetables like *matsutake* mushroom and bamboo shoots. Moreover, various cooking methods, like deep-frying (*tempura*), are applied to enjoy the texture of such seasonal foods.^37^ Seasonal differences in AOC values were observed in foods typically denoted as seasonal products, suggesting that foods exhibiting high AOC were partially influenced by the harvest season as well as the method of preparation popular in a given season.

In the present study, L-ORAC values were generally low, approximately 1/10 that of H-ORAC. In general, the more energy you consume, the more foods and nutrients you consume. This was also confirmed for the ORAC intake presented in Table 4. Even though H-ORAC typically closely reflects the content of total ORAC, this is not true for human plasma determinations. Since the food groups that contribute to H-ORAC and L-ORAC, and the behavior of plasma ORAC levels differed greatly,^19^ it is possible that there are differences in the absorption and metabolism of compounds measured by H- and L-ORAC in the human body. It is also important to consider differences in plant- and animal-derived AOC. Although L-ORAC was relatively low, approximately 1/10 that of H-ORAC, the dietary characteristics of high L-ORAC more appropriately reflected the Japanese-style diet. Lipophilic components might have different functions and/or metabolic pathways in the body because of differences in the physicochemical properties of hydrophilic components,^19^ as well as fat-^38^ and water-soluble vitamins and minerals.^39^ It is thought that excess intake of water-soluble vitamins is excreted rapidly in the urine, whereas fat-soluble vitamins are stored in the body and used for metabolizing over long periods. A similar phenomenon had also been observed in both plasma H- and L-ORAC concentration after the meal.^19^ These differences may have resulted in the outcome of disease prevention only by the use of the lipophilic antioxidant supplement in the previous study.^20^ Further studies to generate additional data regarding *in vivo* mechanisms are needed to clarify these points.

The results of this study should be interpreted cautiously. First, the processing of foods by cooking and the various pathways of food digestion also affect the nature and molecular structures of the antioxidant compounds.^40^ A previous study indicated that foods with active polyphenolic flavonoids are more resistant to degradation than foods with vitamins and related compounds.^35^ Several *in vitro* studies reported that cooked foods showed comparatively higher ORAC values than raw or uncooked foods (eg, red cabbage [H-ORAC], russet potato [H-ORAC], and tomato [H-/L-ORAC]),^41^ while others showed lower values (eg, carrot [H-/L-ORAC], broccoli [L-ORAC], and russet potato [L-ORAC]). The available data on the effects of processing is limited, and additional data will be needed to reveal differences in processed foods. Removal of the peel is a well-known factor that may influence AOC values of produce.^42^ Nonetheless, a previous study reported that meals high in AOC tended to maintain high AOC levels even following cooking (unpublished data). In this respect, the Japanese diet might be considered optimal for high AOC intake. Second, the present study estimated information of food and nutrient intake on the basis of dietary recall in elderly subjects living in rural areas in 1996–1997. It is possible that this dietary habit does not accurately reflect the current situation, especially in large cities.

The coverage rates among the food groups extremely varied (64.1–96.5%). The diversity of eating habits may be attributed to factors, such as age, gender, region, and social status. The differences in data coverage indicate that food groups with lower weight contribution to AOC intake comprise a greater variety of foods. The food in this study was preferentially selected from high-intake foods using a list ranked according to the amount of individual food items in each food group in the dietary records. The missing values in the present composition database have a minor impact on overall food intake due to minorities in terms of weight intake. In contrast, this study did not consolidate foods that are similar but have different food numbers; thus, we could not prevent the risk of underestimating the AOC values. In addition to the abovementioned considerations, compared with the food intake in the National Health and Nutrition Survey, Japan in 1996, 1997, and 2017, the intake of fish and shellfish, algae, and dairy products was more and that of meats was less in the present study. Although a direct comparison is not appropriate because of the use of different survey methods and the definition of data collection, the food intakes from the present study are considered to reflect the eating habits of the population in the rural Tohoku region in the north-eastern part of Japan, and these coverages may only be applicable to the population in the Tohoku region. It may be worthwhile to assess the effect of the intakes of each food group on inter-individual variation at an AOC level. Future epidemiological studies may include additional food items according to dietary habits in a certain research population to adequately estimate antioxidant intakes. This ORAC database can be added to the AOC measurement value of other food items according to different regions and eras in Japan without compromising total reliability using the same validated ORAC methods.^28^^,^^29^ Thus, more accurate associations between AOC intake from Japanese diet and disease outcomes can be assessed.

The ORAC method evaluated only one aspect of the antioxidative potential of foods, a number of other chemical techniques, such as Ferric Ion Reducing Antioxidant Power and Trolox Equivalent Antioxidant Capacity, have been developed to measure the AOC of foods.^43^ The AOC values cannot be directly compared between each method because of different mechanisms and different radical or oxidant sources. Though several compounds responsible for ORAC have been identified,^35^ other compounds responsible for the high AOC values are still largely unknown. The utilization of more than one method of AOC determination might enable a more accurate measurement of AOC in the diet.

In conclusion, the present results indicate that a Japanese diet, which is characterized by the high intake of rice, seafood, soybean products, and vegetables, might be an optimal food pattern to achieve high contributions to AOC. To avoid misinterpretation and improper application by consumers of data regarding AOC of foods, further studies are warranted, not only to confirm determinations using validated methods, but also to clarify the relationships between food intake and health outcomes.

## APPENDIX A. SUPPLEMENTARY DATA

eTable 1. Antioxidant capacity of selected foods in Japan (last modified May 8, 2017)

eTable 2. Seasonal variances of antioxidant capacity by food groups in the study participants
