# Supplementary material for: The Major Source of Antioxidants Intake From Typical Diet Among Rural Farmers in North-eastern Japan in the 1990s
Source: J Epidemiol. 2021 Feb 5;31(2):101–8. doi: 10.2188/jea.JE20190237 (PMC7813768; doi:10.2188/jea.JE20190237)
Supplement: Supplementary file 1 [file je-31-101-s001.pdf]

eTable 1. Antioxidant capacity of selected foods in Japan (last modified May 8, 2017)

Food groups	Food item number[a]	Food and description	H-ORAC (μmol TE/g edible portion)					L-ORAC (μmol TE/g edible portion)				
			N	Mean	SD	Min	Max	N	Mean	SD	Min	Max
Rice, bread, and noodles	01015	Wheat, soft flour, first grade	2	14.55	—	10.50	18.61	2	6.11	—	3.96	8.25
	01026	Wheat, white table bread	2	6.19	—	5.79	6.59	2	0.91	—	NQ	1.82
	01031	Wheat, French bread	2	7.09	—	6.29	7.88	2	NQ	—	—	—
	01034	Wheat, soft rolls	2	8.42	—	7.65	9.18	2	2.39	—	2.10	2.68
	01039	Wheat, udon, wet form, boiled	2	1.95	—	1.46	2.44	2	NQ	—	NQ	NQ
	01044	Wheat, somen and hiyamugi, dry form, bioled	2	3.91	—	3.48	4.33	2	NQ	—	—	—
	01048	Chinese noodles, wet-form, boiled	2	5.40	—	3.68	7.13	2	NQ	—	NQ	NQ
	01064	Macaroni and spaghetti, dry-form, boiled	2	4.34	—	3.83	4.86	2	NQ	—	NQ	NQ
	01085	Cooked paddy rice, brown rice	2	4.49	—	4.30	4.68	2	3.14	—	2.03	4.25
	01088	Cooked paddy rice, well-milled rice	2	2.04	—	2.01	2.08	2	NQ	—	NQ	NQ
	01118	Glutinous rice products, sekihan	2	3.25	—	3.05	3.44	2	1.21	—	0.37	2.06
	01128	Buckwheat noodle, wet form, boiled	2	12.78	—	11.65	13.90	2	0.44	—	NQ	0.88
Potatoes	02003	Konjac, block, made from fine powder	2	0.23	—	NQ	0.47	2	NQ	—	NQ	NQ
	02006	Sweet potato, tuberous root, raw	3	6.26	1.03	5.28	7.33	3	1.15	0.44	0.69	1.57
	02010	Taro (satoimo), corm, raw	3	10.10	4.14	7.15	14.83	2	0.75	—	0.58	0.91
	02017	Potato, tuber, raw	4	6.61	0.92	5.53	7.72	3	0.86	0.27	0.56	1.08
	02023	Chinese yam (nagaimo), tuberous root, raw	2	3.76	—	3.29	4.22	2	0.59	—	0.20	0.97
Sugars	03001	Brown sugar lump	2	32.57	—	29.70	35.44	2	0.67	—	NQ	1.33
	03003	Soft sugar, white	2	NQ	—	NQ	NQ	2	NQ	—	NQ	NQ
	03004	Soft sugar, yellow	2	NQ	—	NQ	NQ	2	NQ	—	NQ	NQ
	03005	Hard sugar, granulated sugar	2	NQ	—	NQ	NQ	2	NQ	—	NQ	NQ
	03022	Honey	2	NQ	—	NQ	NQ	—	ND	—	—	—
Beans	04004	Adzuki bean, bean paste (an), koshi-an	2	5.31	—	2.15	8.48	2	NQ	—	NQ	NQ
	04006	Adzuki bean, bean paste (an), tsubushi-an	2	5.69	—	3.16	8.23	2	NQ	—	NQ	NQ
	04009	Kidney beans, Uzura-mame	2	10.82	—	8.23	13.40	2	1.66	—	1.11	2.21
	04024	Soybean, whole bean, domestic, dried, boiled	2	16.23	—	12.30	20.16	2	2.79	—	2.24	3.34
	04032	Soybean, momen-tofu	3	6.85	1.57	5.34	8.47	2	0.89	—	0.86	0.91
	04033	Soybean, kinugoshi-tofu	2	5.83	—	5.36	6.29	2	0.93	—	0.57	1.29
	04040	Soybean, abura-age	2	10.68	—	8.50	12.85	2	9.68	—	3.72	15.64
	04046	Soybean, itohiki-natto	2	50.84	—	38.19	63.49	2	5.08	—	4.61	5.54
	04051	Okara[2)], Modern product	2	6.60	—	5.31	7.89	2	0.66	—	0.50	0.81
Nuts and seeds	05002	Almond, oil-roasted and salted	2	16.13	—	15.29	16.97	2	2.55	—	2.40	2.69
	05005	Cashew nut, oil-roasted and salted	2	13.17	—	12.39	13.95	2	10.60	—	4.82	16.38
	05014	Walnut, roasted	2	65.82	—	63.82	67.81	2	5.26	—	4.85	5.66
	05018	Sesame seed, roasted	2	26.73	—	26.64	26.82	2	23.68	—	12.42	34.94
	05035	Peanut, roasted	2	28.03	—	26.44	29.62	2	4.34	—	3.49	5.19
	05036	Peanut, oil-roasted and salted	2	25.84	—	24.62	27.07	2	3.79	—	2.68	4.89
Vegetables	06015	Edamame, raw	3	12.30	3.09	8.82	14.75	2	4.49	—	4.16	4.82
	06046	Pumpkin (Cucurbita moschata), fruit, raw	3	3.28	0.56	2.78	3.88	3	1.94	0.99	0.87	2.83
	06061	Cabbage, head, raw	5	3.72	0.79	2.40	4.46	4	0.77	0.46	0.23	1.30
	06065	Cucumber, fruit, raw	30	1.93	0.32	1.53	2.66	28	1.25	0.23	0.75	1.82
	06066	Cucumber, salted pickles	2	1.66	—	1.65	1.67	2	0.88	—	0.78	0.98
	06084	Edible burdock, root, raw	3	66.07	12.74	52.17	77.19	2	1.56	—	1.41	1.70
	06086	Komatsuna, leaves, raw	34	12.58	4.22	5.17	23.12	34	4.24	1.28	1.87	8.28
	06099	Garland chrysanthemum, leaves, raw	19	46.44	18.57	17.80	96.03	19	6.78	1.45	4.43	9.83
	06134	Japanese radish (daikon), root without skin, raw	3	3.88	0.92	2.93	4.78	2	0.17	—	0.17	0.18
	06139	Japanese radish (daikon), takuan-zuke	2	4.77	—	3.82	5.73	2	2.86	—	2.21	3.50
	06151	Bamboo, shoot, canned in water	4	4.10	3.15	1.44	8.39	2	3.25	—	1.46	5.04
	06153	Onion, bulb, raw	18	9.31	2.05	6.28	13.17	17	0.23	0.12	0.08	0.51
	06175	Sweet corn, immature kernels, raw	16	4.83	0.65	3.88	6.29	16	2.15	0.88	1.25	4.78
	06182	Tomato, fruit, raw	32	3.32	0.45	2.64	4.92	31	0.41	0.10	0.16	0.63
	06185	Tomato, juice	2	5.61	—	3.59	7.63	—	ND	—	—	—
	06191	Eggplant, fruit, raw	3	27.65	11.65	15.55	38.79	2	0.58	—	0.14	1.02
	06201	Turnip rape, flower buds and stems, raw	1	44.48	—	—	—	1	7.82	—	—	—
	06207	Chinese chive, leaves, raw	2	12.31	—	11.83	12.79	2	4.52	—	3.59	5.45
	06212	Carrot, root with skin, raw	3	3.36	1.90	1.46	5.26	2	0.74	—	0.46	1.02
	06226	Welsh onion (nebuka-negi), leaves, blanched, raw	3	2.61	0.26	2.32	2.79	2	0.51	—	0.48	0.55
	06233	Chinese cabbage, head, raw	3	2.80	0.74	1.96	3.33	2	0.32	—	0.26	0.37
	06236	Chinese cabbage pickles, kim chee	2	6.19	—	5.71	6.68	2	2.68	—	1.67	3.68
	06245	Green sweet pepper, fruit, raw	3	8.64	2.76	6.95	11.83	2	1.15	—	1.10	1.20
	06263	Broccoli, inflorescence, raw	3	16.10	4.41	11.00	18.74	3	3.04	1.84	1.41	5.05
	06267	Spinach, leaf, raw	37	18.90	5.31	7.29	33.03	34	7.92	2.08	5.45	13.87
	06291	Mung bean sprout, raw	3	5.93	0.32	5.57	6.17	2	0.54	—	0.52	0.56
	06312	Crisp lettuce, head, raw	4	2.12	0.86	1.09	3.02	2	0.56	—	0.53	0.59
	06317	East Indian lotus root, rhizome, raw	3	22.78	3.94	19.57	27.18	2	1.38	—	1.03	1.73
	06325	Bracken, young shoots, raw	2	10.44	—	10.25	10.63	2	7.07	—	5.36	8.77
	07012	Strawberry, raw	4	30.72	6.15	22.45	36.30	2	1.84	—	1.21	2.47

eTable 1. Antioxidant capacity of selected foods in Japan (last modified May 8, 2017)

Food groups	Food item number[a]	Food and description	H-ORAC (μmol TE/g edible portion)					L-ORAC (μmol TE/g edible portion)				
			N	Mean	SD	Min	Max	N	Mean	SD	Min	Max
Fruits	07028	Satsuma mandarin, juice sac, early ripening type, raw	3	13.57	0.82	12.93	14.49	2	0.11	—	NQ	0.22
	07040	Navel, Juice sacs, raw	2	14.51	—	6.15	22.88	2	NQ	—	NQ	NQ
	07041	Valencia orange, juice sac, raw	3	20.13	1.16	18.91	21.21	2	0.43	—	0.41	0.46
	07049	Japanese persimmon (kaki), nonastringent, raw	3	5.64	1.65	4.62	7.54	2	NQ	—	NQ	NQ
	07054	Kiwifruit, raw	3	7.71	1.28	6.45	9.02	2	0.47	—	0.45	0.49
	07062	Grapefruit, juice sac, raw	3	18.13	2.80	15.89	21.28	2	0.45	—	0.44	0.46
	07063	Grapefruit, straight fruit juice	2	13.07	—	10.28	15.86	—	ND	—	—	—
	07064	Grapefruit, reconstituted fruit juice	2	10.30	—	10.08	10.52	—	ND	—	—	—
	07077	Watermelon, raw	3	1.87	0.10	1.79	1.98	2	0.08	—	NQ	0.16
	07088	Japanese pear, raw	2	1.59	—	1.28	1.89	2	0.08	—	NQ	0.16
	07091	European pears, Raw	2	3.40	—	3.05	3.75	2	0.18	—	NQ	0.37
	07097	Pineapple, raw	3	6.30	0.30	6.03	6.63	3	0.42	0.37	NQ	0.70
	07099	Pineapple, reconstituted fruit juice	2	6.28	—	5.56	7.01	—	ND	—	—	—
	07107	Banana, raw	3	7.48	2.03	5.14	8.69	2	1.14	—	1.08	1.19
	07116	Grape, raw	3	3.88	0.57	3.23	4.21	2	NQ	—	NQ	NQ
	07119	Grape, reconstituted fruit juice	2	24.78	—	13.51	36.05	—	ND	—	—	—
	07135	Melon, open culture, raw	3	2.55	0.60	1.86	2.99	2	0.09	—	NQ	0.18
	07136	Peach, raw	2	23.28	—	17.96	28.59	2	0.71	—	0.68	0.73
	07148	Apple, raw	2	18.02	—	16.89	19.14	2	0.32	—	0.30	0.33
	07149	Apple, straight fruit juice	2	16.99	—	15.55	18.43	—	ND	—	—	—
	07150	Apple, reconstituted fruit juice	2	3.63	—	1.34	5.92	—	ND	—	—	—
Mushrooms	08001	Winter mushroom, raw	2	7.02	—	6.25	7.79	2	1.80	—	1.68	1.92
	08011	Shiitake mushroom, raw	3	2.86	0.25	2.58	3.06	2	1.98	—	1.58	2.39
	08013	Shiitake, Hoshi-shiitake	2	24.99	—	24.68	25.29	2	15.17	—	9.68	20.66
	08016	Bunashimeji, raw	2	4.23	—	3.63	4.82	2	3.78	—	3.49	4.07
	08028	Maitake, raw	2	4.09	—	3.99	4.19	2	1.28	—	1.16	1.40
Algae	09004	Purple laver, toasted	2	209.79	—	161.19	258.38	2	12.40	—	11.89	12.91
	09017	Ma-kombu, dried	5	35.53	29.38	4.50	68.49	5	9.12	2.42	5.53	12.36
	09018	Mitsuishi-kombu, dried	2	4.07	—	1.88	6.26	2	29.88	—	22.43	37.33
	09023	Kombu, tsukudani	2	20.09	—	17.69	22.50	2	0.66	—	NQ	1.31
	09031	Hijiki, boiled and dried	2	14.65	—	11.40	17.91	2	14.16	—	13.44	14.89
	09038	Mozuku, salted, desalted	2	0.56	—	0.28	0.84	2	0.69	—	0.46	0.92
	09045	Wakame, blanched and salted, desalted	3	2.37	0.41	1.92	2.74	3	2.32	2.41	NQ	4.81
Fish and shellfish	10003	Horse mackerel, raw	2	4.95	—	4.62	5.29	2	6.45	—	4.53	8.38
	10045	Japanese anchovy, niboshi	2	20.99	—	20.10	21.89	2	30.99	—	22.00	39.99
	10070	Japanese eel, kabayaki	2	10.70	—	10.30	11.10	2	3.00	—	2.18	3.82
	10087	Skipjack, caught in autumm,raw	1	11.04	—	—	—	1	9.35	—	—	—
	10100	Brown sole, raw	2	4.23	—	3.23	5.24	2	5.89	—	3.01	8.77
	10134	Salmon, chum salmon, raw	2	7.58	—	7.11	8.06	2	5.96	—	5.93	5.98
	10140	Chum salmon, salted roe (ikura)	1	24.64	—	—	—	1	15.54	—	—	—
	N/A	Chum salmon, roe (ikura), marinated in soy sauce (ikura	1	45.81	—	—	—	1	4.38	—	—	—
	10154	Mackerel, raw	2	6.42	—	5.94	6.89	2	5.63	—	2.99	8.26
	10173	Pacific saury, raw	2	8.47	—	8.25	8.69	2	9.78	—	7.96	11.59
	10193	Red sea bream, cultured, raw	2	4.62	—	4.26	4.99	2	3.58	—	2.09	5.08
	10202	Walleye pollack, roe (tarako), raw	2	22.12	—	17.99	26.25	2	28.39	—	27.48	29.30
	10205	Pacific cod, raw	2	3.97	—	3.64	4.31	2	6.97	—	4.18	9.76
	10252	Yellowfin tuna, raw	2	8.98	—	8.73	9.23	2	2.44	—	2.36	2.52
	10253	Bluefin tuna, Lean meat, raw	2	7.60	—	6.96	8.24	2	3.24	—	2.32	4.15
	10255	Albacore, raw	1	8.29	—	—	—	1	2.39	—	—	—
	10281	Short-necked clams, raw	2	8.57	—	7.93	9.22	2	4.76	—	3.90	5.62
	10321	Tiger prawn, cultured, raw	2	3.57	—	2.52	4.62	2	3.47	—	2.83	4.12
	N/A	Argentine red shrimp, raw	1	8.73	—	—	—	1	6,96	—	—	—
	10345	Japanese common squid (surumeika), raw	2	5.61	—	4.70	6.52	2	12.14	—	9.40	14.88
	10380	Fish paste product, yakinuki-kamaboko[3)]	2	3.30	—	2.95	3.65	2	0.17	—	NQ	0.35
	10381	Fish paste product, yaki-chikuwa[4)]	2	6.01	—	4.40	7.62	2	0.87	—	0.51	1.24
	10388	Fish paste product, fish sausage	2	3.63	—	3.18	4.07	2	1.63	—	0.95	2.30
Meats	11030	Cattle, chuck, lean and fat, raw	2	4.90	—	4.80	5.00	2	2.46	—	2.34	2.58
	11046	Cattle, flank or short plate, lean and fat, raw	2	4.59	—	4.23	4.95	2	3.28	—	3.18	3.38
	11075	Imported beef, inside round, lean and fat, raw	2	6.32	—	4.67	7.97	2	2.70	—	2.35	3.05
	11130	Pork, large type breeds, inside ham, lean and fat, raw	4	5.53	0.07	5.46	5.63	4	2.69	0.41	2.35	3.29
	11149	Pork, large type breeds, loin, lean and fat, raw	2	5.58	—	5.20	5.96	2	2.66	—	1.70	3.62
	11163	Swine, ground meat, raw	2	5.47	—	5.01	5.94	2	3.32	—	2.48	4.17
	11166	Pork, medium type breeds, liver, raw	2	12.41	—	12.19	12.64	2	8.57	—	8.00	9.14
	11176	Pork, ham made from loin	2	7.35	—	6.78	7.93	2	1.53	—	1.47	1.60
	11186	Pork, Vienna sausage	2	6.44	—	5.58	7.30	2	8.11	—	7.80	8.42
	11219	Chicken, broiler, breast, meat with skin, raw	2	10.29	—	9.35	11.23	2	1.21	—	0.77	1.66
	11220	Chicken, broiler, breast, meat without skin, raw	2	10.16	—	9.84	10.47	2	1.19	—	1.11	1.27

eTable 1. Antioxidant capacity of selected foods in Japan (last modified May 8, 2017)

Food groups	Food item number[a]	Food and description	H-ORAC (µmol TE/g edible portion)					L-ORAC (µmol TE/g edible portion)				
			N	Mean	SD	Min	Max	N	Mean	SD	Min	Max
	11221	Chicken, broiler, thigh with skin, raw	4	7.17	0.85	6.06	8.12	4	3.59	2.58	1.63	7.33
	11224	Chicken, broiler, thigh, meat without skin, raw	2	7.98	—	7.47	8.50	2	4.49	—	1.30	7.68
	11232	Chicken, offal and by-products, liver, raw	2	10.79	—	7.56	14.02	2	6.56	—	6.51	6.62
Eggs	12004	Hen's egg, whole, raw	2	8.03	—	7.23	8.83	2	1.78	—	1.69	1.88
	12005	Hen's egg, whole, boiled	2	5.65	—	5.03	6.27	2	1.79	—	1.44	2.14
	12017	Hen's egg product, tamago-dofu	2	2.74	—	2.47	3.02	2	0.80	—	0.79	0.81
	12018	Hen's egg product, rolled omelette (atsuyakitamago)	2	5.33	—	4.46	6.21	2	1.75	—	1.35	2.15
Dairy products	13003	Ordinary liquid milk	2	0.86	—	NQ	1.72	—	ND	—	—	—
	13005	Milk containing recombined milk, low fat	2	5.64	—	NQ	11.27	—	ND	—	—	—
	13007	Milk beverage, coffee flavored	2	8.48	—	5.24	11.72	—	ND	—	—	—
	13025	Yogurt, whole milk, unsweetened	3	1.13	0.14	0.96	1.21	3	0.22	0.19	NQ	0.37
	13026	Yogurt, skimmed, sweetened	2	1.05	—	0.97	1.14	2	NQ	—	NQ	NQ
	13040	Process cheese	2	13.42	—	11.98	14.85	2	1.05	—	0.73	1.37
	13043	Ice cream, regular fat	2	3.35	—	2.05	4.65	2	3.00	—	0.66	5.33
Fats and oils	14006	Vegetable oil, blend	2	0.60	—	0.57	0.64	2	NQ	—	NQ	NQ
	14017	Salted butter	2	0.80	—	0.77	0.84	2	NQ	—	NQ	NQ
	14020	Margarine, soft type	2	1.07	—	1.00	1.13	2	NQ	—	NQ	NQ
Confectioneries	15009	Kasutera	2	8.74	—	8.67	8.81	2	10.67	—	8.83	12.51
	15027	Dorayaki[5)]	2	10.22	—	9.20	11.24	2	3.32	—	3.17	3.46
	15033	Manju Mushi-manju	2	11.15	—	10.16	12.14	2	NQ	—	NQ	NQ
	15069	Bean jam bun	2	9.62	—	8.16	11.08	2	2.81	—	2.16	3.47
	15070	Custard cream bun	2	7.29	—	7.14	7.45	2	1.79	—	1.76	1.81
	15071	Strawberry jam bun	2	9.74	—	9.69	9.78	2	1.74	—	1.59	1.89
	15075	Short cake	2	5.16	—	4.97	5.35	2	3.56	—	3.06	4.06
	15077	Doughnut, yeast-leavened	2	6.15	—	4.25	8.06	2	2.84	—	2.09	3.58
	15086	Custard pudding	2	5.44	—	4.25	6.64	2	10.84	—	5.47	16.20
	15087	Jellies, orange	2	1.99	—	0.96	3.01	2	NQ	—	NQ	NQ
	15104	Potato chips, fabricated	2	18.21	—	12.41	24.01	2	2.06	—	NQ	4.13
	15116	Milk chocolate	2	87.72	—	65.88	109.56	2	21.78	—	18.94	24.63
Beverages	16003	Sake, honjozo	2	2.18	—	1.89	2.48	—	ND	—	—	—
	16006	Beer, pale	2	5.58	—	4.77	6.39	—	ND	—	—	—
	16011	Wine, red	2	29.21	—	25.27	33.15	—	ND	—	—	—
	16014	Distilled through a continuous still	2	NQ	—	NQ	NQ	—	ND	—	—	—
	16037	Green tea, sencha, infusion	2	9.60	—	8.96	10.23	—	ND	—	—	—
	16039	Ban-cha, infusion	2	15.04	—	8.22	21.85	—	ND	—	—	—
	16042	Oolong tea, infusion	3	8.36	1.04	7.40	9.46	—	ND	—	—	—
	16044	Black tea, infusion	2	10.04	—	8.02	12.07	—	ND	—	—	—
	16045	Coffee, infusion	2	35.80	—	33.47	38.12	—	ND	—	—	—
	16047	Coffee drink containing milk	2	36.57	—	20.93	52.22	—	ND	—	—	—
	16053	Cola drink	3	0.25	0.44	NQ	0.76	—	ND	—	—	—
	16055	Mugi-cha, infusion	1	1.22	—	—	—	—	ND	—	—	—
Seasonings and spices	17001	Worcester sauce	2	13.67	—	6.50	20.84	2	1.57	—	0.81	2.33
	17002	Japanese Worcester sauce, semi-thick type	2	20.05	—	14.97	25.13	2	0.80	—	NQ	1.61
	17007	Koikuchi-shoyu	2	52.26	—	49.95	54.58	—	ND	—	—	—
	17015	Grain vinegar	2	0.82	—	NQ	1.63	—	ND	—	—	—
	17030	Seasoned soy saue (mentsuyu), triple strength	2	16.21	—	15.13	17.30	—	ND	—	—	—
	17036	Tomato ketchup	2	5.43	—	2.17	8.70	2	0.92	—	0.59	1.25
	17042	Mayonnaise, whole egg type	2	0.84	—	NQ	1.68	2	2.23	—	1.29	3.17
	17043	Mayonnaise, egg yolk type	2	3.46	—	2.11	4.81	2	3.34	—	1.76	4.92
	17044	Rice-koji miso, sweet type	1	21.58	—	—	—	1	2.26	—	—	—
	17045	Rice-koji miso, light yellow type	2	32.74	—	20.21	45.26	2	6.88	—	NQ	13.76
	17046	Rice-koji miso, red type	2	39.98	—	37.64	42.33	2	17.94	—	14.15	21.73
Prepared foods	18001	Curry, beef, retort-pouched	2	7.68	—	6.16	9.21	2	2.06	—	1.99	2.13
	18002	Chio tzu, frozen	2	7.12	—	5.01	9.23	2	1.80	—	1.51	2.10
	18006	Croquette, cream type, for frying, frozen	2	3.46	—	3.44	3.48	2	1.17	—	0.94	1.40
	18007	Croquette, potato type, for frying, frozen	2	7.67	—	7.45	7.89	2	2.11	—	1.28	2.94
	18013	Hamburg steak, frozen	2	8.99	—	8.60	9.37	2	2.21	—	1.60	2.81

ND, not determined; NQ, not quantitated; N/A, not applicable ; SD, standard deviation

[a.] Item numbers were addressed under the Food Composition Table 2010.

[b] Insoluble residue from soy milk processing.

[c] Baked kamaboko, which is made from pureed white fish.

[d] Baked tubular kamaboko, which is made from pureed white fish.

[e.] A pair of baked round sweet dough filled with An (adzuki bean paste).

DISCLAIMER: This database is for research purposes only, and health claims should not be made based on this database alone. Although we use our best efforts to obtain and publish reliable data, we will not hold liable as to the validity, correctness, accuracy, completeness and/or reliability of this data. In addition, we assume no liability whatsoever associated with the use or misuse of this database.

eTable 2. Seasonal variances of antioxidant capacity by food groups in the study participants

	Food intake (mean, g/day)					H-ORAC (mean, µmol TE/day)					L-ORAC (mean, µmol TE/day)				
Food groups	Spring	Summer	Autumn	Winter	*P* -value[a]	Spring	Summer	Autumn	Winter	*P* -value[a]	Spring	Summer	Autumn	Winter	*P* -value[a]
Rice, bread, and noodles	553.9	537.1	521.6	522.3	*0.012*	1248.0	1241.3	1135.5	1118.7	*0.001*	42.1	42.0	35.5	34.3	*0.345*
Potatoes	41.5	49.5	68.2	61.4	<*.001*	271.2	293.9	418.2	316.4	<*.001*	35.5	39.5	51.3	41.9	<*.001*
Sugars	10.5	10.0	9.8	9.0	*0.345*	7.2	1.5	2.5	3.0	*0.350*	0.1	0.0	0.1	0.1	*0.350*
Beans	87.0	84.3	89.4	88.6	*0.770*	1285.6	1322.2	1264.5	1248.9	*0.928*	224.0	215.6	225.9	214.4	*0.800*
Nuts and seeds	2.8	1.1	2.8	4.7	<*.001*	229.2	132.5	207.8	318.8	*0.015*	61.1	51.9	53.2	72.4	*0.169*
Vegetables	256.0	363.0	247.7	199.5	<*.001*	1045.8	1800.2	1556.7	1261.4	<*.001*	156.9	153.0	163.7	155.9	*0.884*
Fruits	96.3	143.4	183.3	111.1	<*.001*	1696.0	1234.1	1555.8	2016.9	*0.012*	72.8	43.7	41.5	69.7	<*.001*
Mushrooms	10.8	6.3	15.8	13.4	<*.001*	108.4	91.8	116.5	102.4	<*.001*	54.5	43.2	53.2	47.1	*0.002*
Algae	16.5	13.5	10.1	14.0	*0.006*	207.7	179.7	182.7	216.1	*0.569*	54.1	55.1	39.8	62.1	*0.370*
Fish and shellfish	119.7	123.6	135.6	128.4	*0.075*	557.9	757.2	495.5	501.2	<*.001*	498.7	710.0	477.0	469.5	<*.001*
Meats	44.0	42.5	45.2	45.8	*0.818*	234.7	264.2	304.3	226.9	*0.008*	137.3	132.7	158.8	114.8	*0.031*
Eggs	48.7	43.3	44.7	42.7	*0.063*	454.9	435.7	422.9	419.2	*0.255*	102.5	99.0	96.6	94.7	*0.309*
Dairy products	163.6	171.3	150.6	149.6	*0.056*	343.1	430.0	306.0	340.1	*0.001*	11.1	16.9	4.7	4.3	*0.053*
Fat and oil	9.1	9.9	9.1	8.9	*0.359*	6.2	6.5	6.8	6.2	*0.057*			NQ		
Confectioneries	31.8	27.6	35.8	34.2	*0.120*	607.5	444.8	724.7	1053.1	<*.001*	191.7	133.8	183.2	324.0	<*.001*
Beverages	717.5	701.4	683.9	700.4	*0.791*	6681.0	5894.9	6802.8	6822.3	*0.084*			ND		
Seasonings and spices	69.1	67.4	62.9	68.7	*0.190*	830.8	804.9	787.7	832.7	*0.537*	297.9	296.2	291.5	297.6	*0.980*
Prepared foods	4.2	5.3	7.8	5.4	*0.469*	518.6	427.9	546.9	672.3	*0.896*	133.3	111.2	142.0	175.3	*0.883*
Total	2282.9	2400.5	2324.3	2208.2	<*.001*	13109.4	12947.0	13668.2	13286.9	*0.330*	1344.9	1375.7	1347.5	1359.8	*0.930*

H-ORAC, hydrophilic oxygen radical absorbance capacity; L-ORAC, lipophilic ORAC.

[a] Obtained using generalized linear model for repeated measures
